# Spatial correlations between browsing on balsam fir by white‐tailed deer and the nutritional value of neighboring winter forage

**DOI:** 10.1002/ece3.3878

**Published:** 2018-02-10

**Authors:** Emilie Champagne, Ben D. Moore, Steeve D. Côté, Jean‐Pierre Tremblay

**Affiliations:** ^1^ Département de biologie Centre d’études nordiques & Chaire de recherche industrielle CRSNG en aménagement intégré des ressources de l’île d'Anticosti Université Laval Québec Canada; ^2^ Centre d’étude de la forêt Université Laval Québec Canada; ^3^ Hawkesbury Institute for the Environment Western Sydney University Richmond NSW Australia

**Keywords:** cervid, chemical composition, deer browsing, neighborhood effect, nutritional value, spatial analysis

## Abstract

Associational effects, that is, the influence of neighboring plants on herbivory suffered by a plant, are an outcome of forage selection. Although forage selection is a hierarchical process, few studies have investigated associational effects at multiple spatial scales. Because the nutritional quality of plants can be spatially structured, it might differently influence associational effects across multiple scales. Our objective was to determine the radius of influence of neighbor density and nutritional quality on balsam fir (*Abies balsamea*) herbivory by white‐tailed deer (*Odocoileus virginianus*) in winter. We quantified browsing rates on fir and the density and quality of neighboring trees in a series of 10‐year‐old cutovers on Anticosti Island (Canada). We used cross‐correlations to investigate relationships between browsing rates and the density and nutritional quality of neighboring trees at distances up to 1,000 m. Balsam fir and white spruce (*Picea glauca*) fiber content and dry matter *in vitro* true digestibility were correlated with fir browsing rate at the finest extra‐patch scale (across distance of up to 50 m) and between cutover areas (300–400 m). These correlations suggest associational effects, that is, low nutritional quality of neighbors reduces the likelihood of fir herbivory (associational defense). Our results may indicate associational effects mediated by intraspecific variation in plant quality and suggest that these effects could occur at scales from tens to hundreds of meters. Understanding associational effects could inform strategies for restoration or conservation; for example, planting of fir among existing natural regeneration could be concentrated in areas of low nutritional quality.

## 1 INTRODUCTION

The average rates of herbivory experienced by species in a plant community provide useful information about herbivore diet composition (Lashley, Chitwood, Street, Moorman, & DePerno, 2016) and the relative preference for plant species (Manly, McDonald, Thomas, McDonald, & Erickson, 2002), but may not predict the fate of each individual plant. Because the quality of individual plants is variable (Moore, Andrew, Külheim, & Foley, 2014) and because herbivores make foraging choices at several spatial scales (Senft et al., 1987), realized consumption is variable among plant parts, individuals, and patches. In the context of increased large herbivore populations throughout the Northern Hemisphere (Côté, Rooney, Tremblay, Dussault, & Waller, 2004; Tape, Gustine, Ruess, Adams, & Clark, 2016), knowledge about the risk of consumption has a practical use for managing specific plant species, both via the identification of refugia and because fine‐scale foraging decisions can generate broad‐scale processes, such as changes in plant population dynamics (Brown & Allen, 1989).

Multiple factors can influence the decision of a herbivore whether or not to eat a plant, including the physiological state of the animal (Lima, 1988; Moore, Wiggins, Marsh, Dearing, & Foley, 2015), the perceived risk of predation at that location (Brown, Laundré, & Gurung, 1999; Kuijper et al., 2013; Lima & Dill, 1990), the nutritional value and defense traits of the plant (Moore, Foley, Wallis, Cowling, & Handasyde, 2005; Pyke, Pulliam, & Charnov, 1977), and the presence, identity, and quality of neighboring plants (associational effects, sensu Bergvall, Rautio, Kesti, Tuomi, & Leimar, 2006). Associational susceptibility is an increase in herbivory caused by the presence of neighboring plants (Atsatt & O'Dowd, 1976; Thomas, 1986) and its inverse, associational defense, is a decrease in herbivory due to neighboring plants (Atsatt & O'Dowd, 1976; Tahvanainen & Root, 1972). For example, the presence of *Carex atherodes* increased the risk of grazing by bison (*Bison bison*) on *Carex aquatilis*, an associational susceptibility that can be explained by higher energy gains for the herbivore when consuming both species of *Carex* (Courant & Fortin, 2010). Swamp wallabies (*Wallabia bicolor*) reduced their search effort in patches of low perceived quality, thereby decreasing herbivory on high‐quality plants in those patches, an example of associational defense (Stutz, Banks, Dexter, & McArthur, 2015). As illustrated by those examples, associational effects are an outcome of forage selection processes.

Forage selection is the process by which herbivores fill their energetic and nutrient requirements while limiting energy expenditure (Pyke et al., 1977; Raubenheimer, Simpson, & Mayntz, 2009) and the ingestion of defense compounds (Bryant & Kuropat, 1980). To achieve this goal, forage selection is performed hierarchically at multiple scales, from the choice of a home range to the choice of a bite taken from a plant (Johnson, 1980). Broader scales of forage selection, and thus habitat selection, are expected to be driven by factors with existential impacts on individual fitness, such as predation risk and the thermal environment, while factors with more incremental impacts, such as forage quality, should influence finer‐scale selection (Rettie & Messier, 2000). In predator‐free systems, the influence of forage quality can often be apparent across multiple spatial scales of selection (Massé & Côté, 2009). A few studies have investigated associational effects in the context of hierarchical forage selection, but most have compared among‐ and within‐patch selection (e.g., Bergvall, Rautio, Siren, Tuomi, & Leimar, 2008; Hester & Baillie, 1998; Hjältén, Danell, & Lundberg, 1993; Huang, Wang, Wang, Li, & Alves, 2012; Rautio, Kesti, Bergvall, Tuomi, & Leimar, 2008; Stutz et al., 2015), although Underwood, Inouye, and Hambäck (2014) proposed that plant populations and landscapes could also generate associational effects. A meta‐analysis, however, indicates that neighboring plants influence forage selection by large mammals over large distances (Champagne, Tremblay, & Côté, 2016). Recently, Moore, Britton, Iason, Pemberton, and Pakeman (2015) described increased grazing by red deer (*Cervus elaphus*) and cattle on protected heathland areas with greater proportions of species‐rich grassland within 1,000 m of the heathland. Browsing on Scots pine (*Pinus sylvestris*) by moose (*Alces alces*) declined with increasing presence of preferred alternative browse at the scale of moose management units (415 km^2^; Herfindal, Tremblay, Hester, Lande, & Wam, 2015). Such studies conducted at large spatial scales, however, are rare and needed to assess the spatial extent of associational effects (Champagne et al., 2016).

Examples of associational effects reported above are linked to the identity and abundance of neighboring heterospecific plants, but intraspecific variation in chemical and nutritional composition of neighboring conspecifics and heterospecifics can produce similar effects (e.g., Bergvall et al., 2006). In natural environments, spatial structure in nutritional quality of a single species has the potential to generate associational effects (Andrew, Peakall, Wallis, & Foley, 2007). Covelo and Gallardo (2004) reported positive spatial autocorrelation in the concentration of polyphenols in *Pinus pinaster* at distances of up to 10 m. Concentrations of several plant secondary metabolites in leaves of *Eucalyptus melliodora* are also spatially autocorrelated at distances up to 40‐60 m (Andrew et al., 2007). Such patterns in the distribution of nutritional quality distribution can influence forage selection by koalas (*Phascolarctos cinereus*), and they are more likely to visit *Eucalyptus* trees surrounded by more palatable neighbors (Moore, Lawler, Wallis, Beale, & Foley, 2010). Although Bergvall et al. (2006) explored quality‐mediated associational susceptibility with captive fallow deer (*Dama dama*), no study has investigated associational effects mediated by intraspecific variation in neighborhood nutritional quality in wild cervids (but see Miller, McArthur, & Smethurst, 2007).

Our objective was to test for and determine the radius of influence of neighbor density and nutritional quality on the extent of browsing on balsam fir (*Abies balsamea*) by white‐tailed deer (*Odocoileus virginianus*) in winter. We were especially interested in associational effects beyond plot or patch scales (>100 m^2^), as such effects have seldom been studied (Champagne et al., 2016), especially in the context of hierarchical forage selection. We hypothesized that neighbor characteristics (i.e., abundance and nutritional quality) partially determine the spatial distribution of browsing on balsam fir; high abundance and/or high quality of neighboring plants should be correlated with high (associational susceptibility) or low (associational defense) browsing on fir. Low nutritional quality of neighboring plants should also be correlated with either high (associational susceptibility) or low (associational defense) browsing on fir. We investigated these relationships on a predator‐free island, where forage selection should mainly be driven by forage characteristics, deer nutritional state, abiotic environmental factors, and locomotion costs (Giroux, Dussault, Tremblay, & Côté, 2016; Massé & Côté, 2009, 2012).

## 2 METHODS

### 2.1 Study area and data collection

We collected data on Anticosti Island (7 943 km^2^) in the Gulf of St. Lawrence, Québec, Canada (49°28′N 63°00′W). The maritime climate is characterized by long and relatively mild winters, with mean January precipitation of 67 mm for the 1995–2015 period (Environment Canada 2016) and approximately one‐third falling as snow (Environment Canada 1982). For the 1995–2005 period, mean annual temperature was 1.9°C (*SD* = 1.6), and mean precipitation 917 mm (*SD* = 131; Environment Canada 2006). Approximately 200 white‐tailed deer were introduced to the island at the end of the 19th century and thrived without natural predators. The population seems to be regulated by the availability of winter forage (Massé & Côté, 2012), and local deer density can exceed 20 deer/km^2^ (Rochette & Gingras, 2007). Heavy deer browsing has modified the ecosystem, from the original forests dominated by balsam fir (Grondin, Berger, Landry, & Leboeuf, 2007) to white spruce‐dominated (*Picea glauca*) stands (Potvin, Beaupré, & Laprise, 2003; Tremblay, Huot, & Potvin, 2006) and open parklands (Barrette, Bélanger, De Grandpré, & Ruel, 2014).

**Figure 1:**
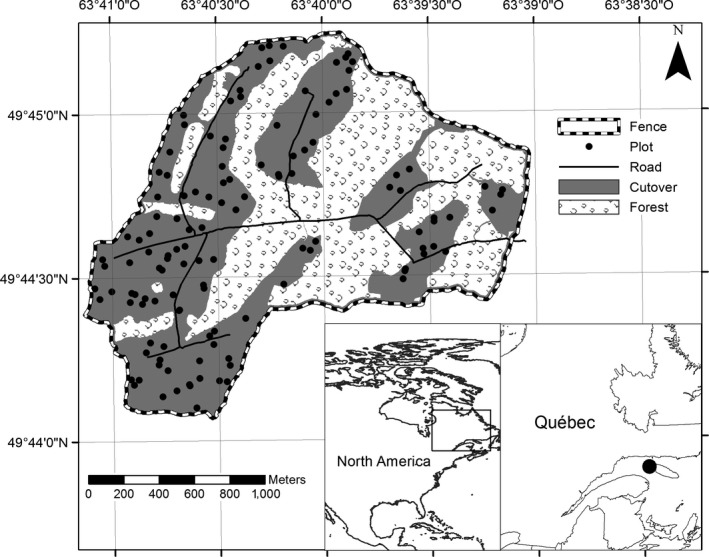
Enclosed study area on Anticosti Island (Québec, Canada). Gray zones are the sampling areas located in the cutover patches, with a 15 m buffer from residual forest patches, fences, and roads. Plots (*n* = 125) were distributed in the sampling area according to a systematic stratified sampling of nonaligned random points, that is, four plots were randomly placed in each cell of a 200 × 200 m grid, with at least 11 m between each to avoid overlap. Each plot consisted of two concentric subplots of 4 and 40 m^2^. Plots located in the sampling area were sampled in 2013 for deer browse (4 m^2^) and abundance of neighboring plants (40 m^2^) and in 2014 for deer browse and nutritional quality (40 m^2^)

We collected data in a 1.4 km^2^ enclosure established in 2000 (Figure 1) as part of the management plan to promote fir regeneration (Beaupré et al., 2004). The enclosure contained mature fir stands and cutover areas harvested in 2000. In the enclosure, deer density was reduced by sport hunting (Côté et al., 2014) and the mean harvest density was 12 deer/km^2^ (*SD* = 10) for the 2000–2012 period (G. Laprise, pers. comm.). Because residual forest patches provided protective cover but little forage for deer, we tallied browsing only in the cutover sections of the enclosure. The cutovers contained regeneration with a mix of balsam fir, white and black spruce (*Picea mariana*), and paper birch (*Betula papyrifera*), with less frequent occurrences of balsam poplar (*Populus balsamifera*), aspen (*Populus tremuloides*), and white pine (*Pinus strobus*). Deer prefer fir to white spruce (Sauvé & Côté, 2007) but deciduous species such as birch and aspen are generally preferred over both spruces and fir, as they have lower concentrations of fiber and are more digestible than conifers (Dumont, Ouellet, Crête, & Huot, 2005). In the first data collection period (25 May 2013–17 June 2013), the enclosure was intact and we measured browsing that had occurred during the 2012–2013 winter. In autumn 2013, breaks were made in the fence in anticipation of the dismantlement of the enclosure. These breaks potentially increased deer density in the enclosure. We measured browsing from winter 2013 to 2014 from 10 to 21 June 2014.

We placed 40 m^2^ circular plots (*n* = 125) throughout the cutover areas according to a systematic stratified sampling design with nonaligned random points (Jensen, 2005). We randomly placed four points in each cell of a 200 × 200 m grid, with at least 11 m between plots to avoid overlap. We established a plot on each point falling in the cutover areas, minus a 15 m buffer from the fence, residual forest patches, and roads (Figure 1).

Each plot consisted of two concentric circular subplots of 4 and 40 m^2^, centered on the fir closest to the random point. In 2013 and 2014, we counted the number of shoots browsed and unbrowsed by deer on palatable species in the 4 m^2^ plot. We counted only shoots that were between 0.25 and 2.25 m in height, because those are considered available for deer during winter (Potvin, 1995). This method mostly captures browsing that occurs in winter, when deer bite shoots. We calculated the number of shoots available per species at the beginning of the winter by summing shoots browsed and unbrowsed. The palatable species found in the plots were birch and fir, with rare balsam poplar, aspen, and white pine. These rare palatable species are not considered further because of their low abundance in the study area. We did not count shoots on white and black spruces, as those are seldom consumed by deer in the presence of alternative resources (Sauvé & Côté, 2007). Unbrowsed shoots were defined as terminal shoots with a minimal length of 5 cm. Snowshoe hares (*Lepus americanus*) were the only other browser present in the study area, and browsing from lagomorphs can be easily differentiated from deer browsing (Potvin, 1995); less than 2% of available shoots were browsed by hares in each year. In 2013, we counted the number of stems of each species taller than 0.11 m in the 40 m^2^ plot. Of those stems, only 6% were shorter than 25 cm and might have been unavailable to browsing during part of the winter. Removing those stems from the statistical analyses does not modify our conclusions. In June 2014, we collected a bulk sample of shoots (length >5 cm) from 3 to 10 different stems in each 40 m^2^ plot for balsam fir, white spruce, and paper birch, when available in the plot (fir = 104 samples, birch = 78, and white spruce = 85). We also collected 124 samples of individual firs and 45 samples of black spruce in the same manner, for a total of 436 samples. These samples were used to develop relationships between near‐infrared spectra (NIRS) and chemical composition (see below). We froze the samples on the day of collection and kept them frozen until drying them in the laboratory.

### 2.2 Chemical analyses and NIRS calibration

Using the samples collected in 2014, we measured several nutritional characteristics (fiber content, digestibility, and nitrogen content) to estimate nutritional quality. Ruminants such as deer generally avoid species rich in fiber, because these compounds reduce forage digestibility and thus nutritional quality (Danell, Bergström, & Edenius, 1994; Danell, Utsi, Palo, & Eriksson, 1994; Forsyth, Richardson, & Menchenton, 2005). In contrast, herbivores prefer plants rich in nitrogen, an essential element in metabolic processes and cellular structure (Mattson, 1980). The bulk shoot samples were oven‐dried at 50°C for 48 hr and milled to pass a 2 mm sieve (Ultra Centrifugal Mill, Type ZM200, RETSCH). All samples were then scanned with a FOSS NIRS DS2500 near‐infrared spectrophotometer (FOSS Analytical A/S, Denmark) between 1,100 and 2 498.2 nm. We performed laboratory analyses on subsets of samples to determine neutral detergent fiber (NDF: hemicellulose, cellulose, and lignin; *n* of subset = 125), acid detergent fiber (ADF: cellulose and lignin; *n* = 126), acid detergent lignin (ADL: lignin; *n* = 126), *in vitro* true digestibility (IVTD_DM_; *n* = 94), and nitrogen content (*n* = 156). We selected the subsets to analyze using the *select* function in WinISI 4.8.0 (FOSS Analytical A/S, Denmark) to cover the range of spectral variation in the population, summarized by a principal components analysis to minimize redundancy in spectra. Fiber fractions were determined with an ANKOM Fibre Analyzer (model 200, ANKOM Technology, NY) and corrected for dry matter content (Goering & Van Soest, 1970). We determined *in vitro* true digestibility on a dry matter basis (IVTD_DM_) using cow ruminal fluid from one fistulated cow kept in an experimental facility (Centre de recherche en sciences animales de Deschambault) and a *Daisy* ^*II*^ Incubator 200 (ANKOM Technology, NY). We followed the filter bag procedure (Operator's manual, *Daisy* ^*II*^ Incubator, ANKOM Technology, NY). We weighed 0.50 ± 0.01 g of ground dry samples into preweighed filter bags (F57, ANKOM size 5 × 5 cm, pore size 25 μm) and placed them in a 5‐L glass jar with the filtered ruminal fluid and a buffer solution under a CO_2_ flow. Jars were kept rotating for 48 hr, at a constant temperature of 39°C. Subsequently, we rinsed the filter bags with water and calculated IVTD_DM_ with the ANKOM procedure (Operator's manual, *Daisy* ^*II*^ Incubator, ANKOM Technology, NY). Cow ruminal fluid is an accepted proxy for white‐tailed deer ruminal fluid in digestion assays (Clemente et al., 2005; Crawford & Hankinson, 1984). Moreover, Jean, Bradley, Berthiaume, and Tremblay (2016) demonstrated that cow ruminal fluid can provide unbiased estimates of digestibility with deer ruminal fluid for balsam fir and white spruce. The inverse of ADF content is another proxy of ruminant digestion, recommended by Jean et al. (2016). By comparing results of both IVTD_DM_ and ADF, our results and conclusions should be robust to bias in digestion and ADF assays (Makkar, Borowy, Becker, & Degen, 1995). Finally, we determined the nitrogen content of the subset of samples using a wet oxidation procedure (Parkinson & Allen, 1975), with a flow injection analyzer (QuikChem 4000 Zellweger Analytics Inc., Lachat Instruments Division, Milwaukee, WI; Diamond, 1992). Prior to the nitrogen analyses, the samples were milled again to pass a 0.5 mm sieve and rescanned with the NIRS.

We developed empirical calibrations for each variable using WinISI 4.8.0 (FOSS Analytical A/S, Denmark) to predict the NDF, ADF, ADL, IVTD_DM_, and nitrogen content of the samples not used in the laboratory analyses (Foley et al., 1998). We included all tree species in the calibrations. Calibrations were developed using modified partial least‐squares regressions with cross‐validation (Shenk & Westerhaus, 1991). We selected the best model that minimized standard error of cross‐validation from candidate models produced with a variety of mathematical treatments applied to the spectra, including the degree of derivatization, smoothing, and scatter correction (DeGabriel, Wallis, Moore, & Foley, 2008). All calibrations were validated using an independent set of 38 samples not used in the calibration. The parameters of the final calibration models are in Appendix S1. In all statistical analyses, we used laboratory values when available.

## 3 SPATIAL ANALYSES

### 3.1 Distribution of fir browsing

To describe the distribution of herbivory in the enclosed area, we tested for spatial autocorrelation in fir browsing rate using a Moran's I correlogram (Moran, 1948), where values above 0 up to 1 indicate positive autocorrelation, and values from 0 to −1 indicate negative autocorrelation. The shape of the correlograms can be visually inspected to detect spatial distribution (patches or gradient); values oscillating around zero indicate random distribution (Fortin & Dale, 2005). We calculated Moran's I values for the browsing rate on firs (number of shoots browsed/number of shoots available) in 2013 and 2014 between all possible pairs of plots, sorted by the distance in meters between the pair (distance classes of 50 m). We considered Moran's I values significant at a level α corrected with a progressive Bonferroni correction (Fortin & Dale, 2005), starting with α = 0.05 and decreasing with cumulative distance classes from 0–50 to 950–1,000 m; α is divided by the rank of the distance class. The chosen bin size of 50 m ensured that every distance class had at least 30 pairs of plots (Fortin & Dale, 2005). The first bin included distance from 11 to 50 m, as plots were separated by a minimum of 11 m to prevent overlap. We considered a maximum distance class of 1,000 m or approximately ½ the length of the side of the study area (Fortin & Dale, 2005). We computed Moran's I with a function provided by G. Larocque (Québec Centre for Biodiversity Science) using R 3.2.1 (R Core Team, 2015).

### 3.2 Correlations between browsing and neighborhood characteristics

We used a two‐step procedure to identify neighborhood characteristics that could explain the spatial distribution of browsing in 2013 and 2014. First, we characterized the spatial distribution of candidate explanatory variables with Moran's I correlograms, using the same procedure as for the browsing rate on fir. We analyzed six variables for each of the three most abundant neighboring plant species (white spruce, balsam fir, and paper birch), for a total of 18 variables: number of individual stems in 40 m^2^ plots and percentages of NDF, ADF, ADL, IVTD_DM_, and nitrogen. We used variables exhibiting significant autocorrelation patterns, based on the significance of the Moran's I value and the correlogram shape (Fortin & Dale, 2005), in the second step of the analysis. We removed variables with a random distribution as our objective was to explain the spatial distribution of browsing by neighboring plant distribution; a random pattern cannot explain the spatial distribution of browsing in the enclosure and appears prone to generating spurious correlations.

In the second step, we investigated the spatial cross‐correlation between browsing rate (2013 and 2014) and the neighborhood characteristics observed to display a spatial pattern in step 1. As we only measured stem abundance in 2013 and nutritional quality in 2014, we correlated 2013 stem abundance and 2014 nutritional quality with browsing in both years. The cross‐correlation method tests the correlation between two variables at multiple distance classes and produces a cross‐correlogram similar to the Moran's I correlogram (Bjørnstad, Ims, & Lambin, 1999; Fortin & Dale, 2005). Positive values in a distance class indicate a positive correlation between the two variables in the pairs of plots in that distance class. Pairs where distance is zero are not included in the analysis, that is, correlation between the two variables within a plot. Correlation is calculated with a centered Mantel statistic in Mantel correlograms. Although the Mantel test has inherent problems with type II error, Mantel correlograms are unaffected by this issue (Legendre, Fortin, & Borcard, 2015). We evaluated the significance of correlation values with *p*‐values generated by 1,000 permutations, and we corrected the α level with a progressive Bonferroni correction. We excluded pairs of plots with missing values from this analysis, such as in the case where no quality values were available because the abundance of the species in the plot was too low. We performed cross‐correlation analyses in R 3.2.1 (R Core Team 2015) using the *correlog* function of the ncf package (Bjørnstad, 2009).

### 3.3 Influence of landscape configuration

Browsing on fir could be more frequent near landscape features used by deer for purposes other than forage selection, such as forested patches used for protective cover against thermal stress and precipitation (Massé & Côté, 2012; Mysterud & Østbye, 1999), and thus influence the results of our previous analyses. We identified three landscape features that could influence deer distribution: (1) the proximity of residual forest patches, used as protective cover by deer (Massé & Côté, 2012); (2) proximity to forest roads not plowed in winter, which are avoided by deer because high snow accumulation increases locomotion costs (Parker, Robbins, & Hanley, 1984); and (3) proximity to fence lines, because of funneling of deer along fences, as suggested by observations of deer trails in enclosed areas. We calculated the distance of each plot to the nearest element of each feature (ArcMAP 10, ESRI 2011). To evaluate the impact of distance to landscape features on browsing rate, we used a generalized linear model (GLM) with a negative binomial distribution, the number of browsed shoots as the response variable and the number of available shoots as a covariate. The explanatory variables were the distances to the fence, to the residual forest, and to the nearest road. We tested a separate model for each year of browsing. We performed the GLM using SAS 9.4 (SAS Institute 2012), with the GENMOD procedure using the type 3 analysis. We report model estimates as estimates [95% confidence intervals].

## 4 RESULTS

### 4.1 Distribution of fir browsing

**Figure 2:**
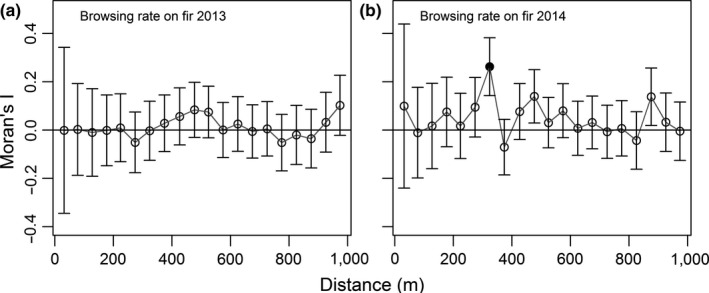
Correlograms for the browsing rate on fir (*Abies balsamea*, number of shoots browsed/number of shoots available) in 4 m^2^ plots in (a) 2013 and (b) 2014 on Anticosti island (Québec, Canada). Moran's I was calculated for pairs of plots in distance classes of 50 m. The first bin included distance from 11 m to 50 m, as plots were separated by a minimum of 11 m to prevent overlap. Error bars are 95% confidence intervals. The black dot indicates a statistically significant value with a progressive Bonferonni correction of the α‐level, starting with α = 0.05

Deer browsing on fir and birch was similarly low in both years of data collection (Table 1). The fir browsing rate was randomly distributed across the study area, based on the Moran's I values (all nonsignificant except one) and on the general shape of the correlogram (Figure 2).

**Table 1:** Descriptive statistics for plot characteristics and variables measured in 4 and 40 m^2^ concentric subplots on Anticosti Island (Québec, Canada). We collected the data for the three species (balsam fir, paper birch, and white spruce) either in 2013 or in 2014. We calculated browsing rate (%) by white‐tailed deer as the number of shoots browsed/number of shoots available in each 4 m^2^ plot. We collected all the other variables in the 40 m^2^ plot. We evaluated fiber content (% NDF: hemicellulose, cellulose, and lignin, ADF: cellulose and lignin, ADL: lignin), *in vitro* dry matter digestibility (IVTD_DM_), and nitrogen content (% N) using bulk samples collected over 3‐10 stems per plot. Distances are expressed in meters

Species	Variables	Year measured	*n*	Mean	Standard deviation
–	Distance to residual forest	–	125	97	82
–	Distance to road	–	125	122	78
–	Distance to fence	–	125	241	159
Balsam fir	Browsing rate	2013	124	5	10
		2014	125	5	8
	Number of stems	2013	125	64	54
	NDF	2014	104	33	2
	ADF	2014	104	27	1
	ADL	2014	104	15.1	0.8
	IVTD_DM_	2014	104	73	1
	N	2014	104	1.1	0.1
Paper birch	Browsing rate	2013	92	18	21
		2014	89	19	24
	Number of stems	2013	125	37	35
	NDF	2014	78	29	3
	ADF	2014	78	29	3
	ADL	2014	78	16	2
	IVTD_DM_	2014	78	75	5
	N	2014	78	2.3	0.3
White spruce	Number of stems	2013	125	25	23
	NDF	2014	85	47	6
	ADF	2014	85	37	5
	ADL	2014	85	20	4
	IVTD_DM_	2014	85	61	5
	N	2014	85	1.0	0.2

### 4.2 Correlations between browsing and neighborhood characteristics

We generated 18 correlograms that describe the spatial autocorrelation structure of the number of individual stems in 40 m^2^ plots and the percentages of NDF, ADF, ADL, IVTD_DM_, and nitrogen for balsam fir, paper birch, and white spruce. Nine correlograms exhibited positive autocorrelations patterns (Appendix S2, Figure S1). The number of individual stems was positively correlated at distances up to 200 m for paper birch and up to 300–350 m for white spruce. The ADF, ADL, and IVTD_DM_ of balsam fir were positively autocorrelated up to 50 m, while NDF, ADF, ADL, and IVTD_DM_ of white spruce were autocorrelated up to 150 m. Other correlograms, especially those for nitrogen content, presented significant Moran’ I values, but not in the first distance classes and surrounding values were not significant, suggesting randomly occurring correlation (Fortin & Dale, 2005).

**Figure 3:**
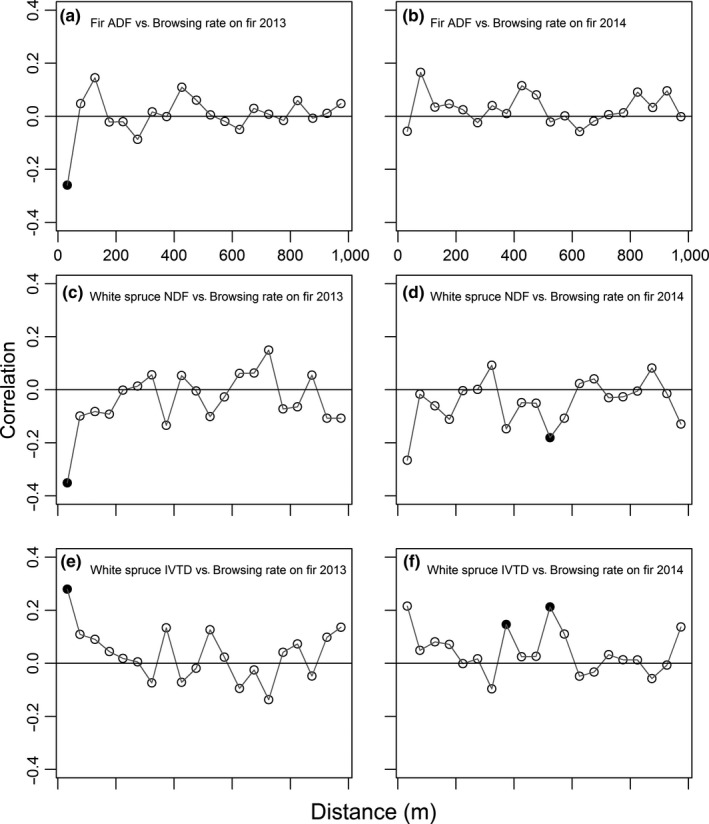
Cross‐correlograms of the correlation between the browsing rate on balsam fir (number of shoots browsed/number of shoots available) in 4 m^2^ plots in 2013 (left column) and 2014 (right column) and (a and b) nutritional characteristics of neighboring fir (*Abies balsamea*) and (c–f) white spruce (*Picea glauca*). Cross‐correlograms between browsing on fir (2013 and 2014) and fir ADL (lignin) content are almost identical to the relation with fir ADF (cellulose and lignin; (g–h) and are presented in Appendix S2. Data were collected on Anticosti island (Québec, Canada). Correlations between each pair of variables were calculated for pairs of plots in distance classes of 50 m, and the point is located at the mean value for the class. The first bin included distance from 11 to 50 m, as plots were separated by a minimum of 11 m to prevent overlap. Black dots indicate statistically significant values with a progressive Bonferonni correction of the α‐level, starting with α = 0.05

We used the nine variables with autocorrelation in their correlogram in cross‐correlation analyses. We tested their correlation with the browsing rate on fir in 2013 and 2014, for a total of 18 cross‐correlograms. Of those, 11 presented at least one significant correlation value. The number of white spruce and birch was not correlated with fir browsing rate in any distance class in either year (Appendix S2, Figure S3). In 2013, fir ADF content was negatively correlated with fir browsing up to 50 m (Figure 3a). Fir ADL displayed an almost identical relation with fir browsing, presented in Appendix S2 (Figure S2) for the sake of brevity. White spruce NDF and ADF contents were also negatively correlated with fir browsing up to 50 m (Figure 3c for NDF, Appendix S2, Figure S2, for ADF), while white spruce IVTD_DM_ was positively correlated with fir browsing in the same distance class (Figure 3e). The cross‐correlograms for the same variables in 2014 had similar shapes, but the values in the first distance class were not significant (Figure 3, right column).

Four cross‐correlograms had significant values of correlation in larger distance classes (between 350 and 600 m), while values in the first distance class (0–50 m) were not significant: white spruce NDF, ADF, ADL, and IVTD_DM_ with fir browsing in 2014 (Figure 3d, f; ADF and ADL: Appendix S2, Figure S2). This repeated pattern could indicate spurious correlations, because they are in contradiction with nearby correlation values, or could indicate a correlation created by differences among cutover patches. The distribution of cutovers included separated or loosely connected patches, separated by approximately 300–400 m of residual forest (Figure 1). To verify that correlations at larger scales were linked to differences among cutover patches, we fitted GLM models with browsing rate of fir in each year, fir IVTD_DM_, and white spruce ADF, ADL, and IVTD_DM_ as response variables. We used cutover patch identity as an independent variable. Browsing rate varied among cutover patches in 2013 but not in 2014 (respectively, *F*_6,117_ = 15.47, *p* = .02; *F*_6,117_ = 8.95, *p* = .18). Values of nutritional characteristics also varied among cutover patches: fir IVTD_DM_ (*F*_6,97_ = 4.14, *p* = .001), white spruce IVTD_DM_ (*F*_5,84_ = 4.06, *p* = .003), and fiber content (NDF: *F*_5,84_ = 4.67, *p* = .0009; ADF: *F*_5,84_ = 6.20, *p* < .0001; ADL: *F*_5,84_ = 4.83, *p* = .0007).

### 4.3 Influence of landscape configuration

As the distance between a plot and the nearest residual forest stand increased, the browsing rate of fir decreased (2013: estimate = −0.005, *F*_1,120_ = 5.73, *p* = .02; 2014: estimate = −0.004, *F*_1,120_ = 5.19, *p* = .02). At the edge of the forest, deer consumed 2% [1, 3] of fir shoots in 2013 and 3% [2, 4] in 2014 while at 100 m from the residual forest patch, they consumed respectively 1% [1, 2] and 2% [2, 3] of fir shoots. The distance to the nearest road did not influence the browsing rate on fir (2013: estimate = −0.0004, *F*_1,120_ = 0.05, *p* = .82; 2014: estimate = 0.001, *F*_1,120_ = 0.33, *p* = .56). The distance to the nearest fence tended toward a negative effect on the browsing rate in 2014, but this decline was imperceptible when comparing browsing rate from the edge to 100 m of a fence line (2013: estimate = 0.0006, *F*_1,120_ = 0.44, *p* = .51; 2014: estimate = −0.002, *F*_1,120_ = 3.54, *p* = .06).

## 5 DISCUSSION

To determine the spatial extent of associational effects beyond a small vegetation patch, we investigated the spatial cross‐correlations between browsing rate on balsam fir by white‐tailed deer and the abundance and nutritional quality of neighboring trees. Browsing on fir was negatively correlated with the fiber content of firs at the finest extra‐patch scale studied (11–50 m). Browsing on fir was also negatively correlated with fiber content and positively correlated with digestibility of white spruce at this scale and inside cutover areas. Recent studies have demonstrated large‐scale associational effects linked to neighboring plant abundance (Herfindal et al., 2015; Moore, Britton, et al., 2015). In contrast to those results and our hypothesis, the abundance of neighboring trees did not generate associational effects, potentially because of the high abundance of resources and the low browsing rate in the study area. Our results, however, suggest that associational effects mediated by the nutritional quality of neighboring conspecific and heterospecific plants can extend to tens to hundreds of meters, in agreement with the hypothesis that associational effects could be applied to entire population or landscape scales (Underwood et al., 2014). Large‐scale associational effects with large herbivores could be the outcome of resource selection at multiple scales or result from a numerical response of the herbivore, through demographic or aggregational processes. Numerical response has been suggested and demonstrated with invertebrate herbivores (Underwood, 2009; Underwood et al., 2014). To date, few studies have investigated associational effects at large scales, in relation with hierarchical forage selection in mammals (Champagne et al., 2016) or in relation to intraspecific variation in plant chemical composition (Miller et al., 2007). Combining these factors seems a promising avenue in understanding how herbivores integrate environmental information from multiple scales while foraging.

Differences in palatability between conspecific plants can generate associational effects (Bee et al., 2009), but most associational effect studies concern interspecific differences in nutritional quality. While differences between heterospecifics are generally larger than between conspecifics (see Table 1), variation in chemical composition or defense traits between conspecifics can be large enough to influence selection by herbivores and thus cause associational effects (Andrew et al., 2007; Sato & Kudoh, 2016). Experimental manipulations of resource nutritional quality can generate associational effects, as Bergvall et al. (2006) and Miller et al. (2007) demonstrated with fallow deer and red‐bellied pademelons (*Thylogale billardierii*). In a natural environment, Moore et al. (2010) demonstrated associational effects caused by intraspecific variation in *Eucalyptus* trees. Our results suggest that unrelated tree species can generate associational effects via nutritional quality.

White‐tailed deer avoid plant species with high fiber content and low digestibility (Dumont et al., 2005; Sauvé & Côté, 2007), as do other deer species (Danell, Bergström, et al., 1994; Forsyth et al., 2005) and as shown by the negative correlation, we report between fir browsing rate and fir fiber content. White spruce has a higher fiber and tannin content than fir and is rarely consumed by deer on Anticosti Island when alternative resources are available (Sauvé & Côté, 2007). Therefore, the negative correlation between fir browsing rate and white spruce fiber content is somewhat surprising and could indicate that white spruce is considered by deer during diet selection, even if it is a low‐quality resource. Consumption of white spruce by deer outside enclosures makes up for 17% of the winter diet (Lefort, Tremblay, Fournier, Potvin, & Huot, 2007), and deer can subsist on a diet with up to 20% of white spruce (Taillon, Sauvé, & Côté, 2006). Deer in enclosed areas might need to consume low‐quality food such as spruce to maintain the optimization of their digestive system for low‐quality diets (Bonin, Tremblay, & Côté, 2016). They could also need to consume spruce to achieve a balance of different nutrients (Raubenheimer et al., 2009) or to avoid overloading a detoxification pathway (Freeland & Janzen, 1974; Marsh, Wallis, Andrew, & Foley, 2006). In all cases, deer could minimize the impact of low‐quality intakes by taking into account intraspecific variation in low‐quality items. Alternatively, white spruce nutritional quality could be indicative of environmental conditions also impacting fir nutritional quality; higher browsing would result from an abundance of nutrients, improving the quality of both spruce and fir. Fir nutritional quality is less variable than white spruce quality (Table 1), which could have prevented the detection of a cross‐correlation with fir browsing. Nevertheless, cross‐correlograms with fir nutritional quality and white spruce nutritional quality present similar shapes. We tested this environmental condition hypothesis by looking at the correlation inside plots between spruce, fir, and birch nutritional quality (Appendix S2). Inside the plots, spruce and fir ADF, ADL, and nitrogen content are correlated, although the other variables of nutritional quality are not. Yet, nutritional characteristics of fir and birch are not correlated and neither are the characteristics of white spruce and birch, at the exception of nitrogen content (Appendix S2), suggesting that plot conditions do not influence all forage types similarly. Further investigation is required beyond the scope of this study to verify whether white spruce quality could be an indicator of unmeasured components of fir quality and explain the link between abiotic conditions and nutritional value.

Multiple variables of nutritional quality were cross‐correlated with fir browsing rates and could have influenced the distribution of fir browsing. The distribution of an ecological variable such as browsing is the result of several processes, intricate and hard to separate (Fortin & Dale, 2005). Some processes have a stronger influence on the ecological variable than other processes and can generate a recognizable spatial pattern. For example, Basque ponies (*Equus ferus*) preferred grass patches, and the distribution of those patches generated a patchy distribution of browse on the less preferred gorse (*Ulex* spp) (Aldezabal, Mandaluniz, & Laskurain, 2012). Contrary to that example, browsing on fir was distributed evenly in the enclosure (Figure 2). The multiple variables cross‐correlated with fir browsing could have had confounding effects on the browse distribution pattern, none however having enough influence to impose its spatial pattern on the browsed shoots distribution pattern. Inside plots, there are few significant correlations between the different species’ nutritional characteristics (Appendix S2), supporting the hypothesis that the patterns of nutritional quality are confounded. It is also possible that such a pattern exists at a smaller scale, but that our study design and the size of our distance classes prevented its detection.

Although the cross‐correlation graphs were very similar between years for each nutritional attribute, fewer correlations were significant in 2014 than in 2013. This result suggests variation in forage selection by deer between the 2 years that could potentially be explained by differences in weather. Cumulative snow cover in 2014 was twice that of 2013, reaching approximately 12,000 day‐cm compared to 6,300 day‐cm (G. Laprise, unpubl. data). Snow accumulation could conceivably allow Anticosti Island deer to reach previously unavailable resources (Potvin, Breton, & Gingras, 1997), but it also more likely renders some smaller fir and neighboring plants completely unavailable. Associational effects are expected to be frequency‐dependent (Underwood et al., 2014) and thereby a modification in the relative availability of tree species could modify their strength.

Distance to the nearest residual forest patch and, to a lesser extent, to the nearest fence also influenced deer browsing rate. The effect size of this relationship was small, but could be more important at higher deer densities. In winter, white‐tailed deer living outside enclosed areas select forest edges, trading‐off locomotion costs against access to forage (Massé & Côté, 2012). Open areas such as cutovers have higher snow accumulation, which increases locomotion costs (Parker et al., 1984), but presents higher forage availability (Massé & Côté, 2012). Consequently, balsam firs located near residual forest patches were more browsed, and browsing decreased on firs further from the forest edge. This edge effect did not bias the correlations we found because the correlations between fir browsing rate and neighboring plant characteristics mostly occurred between plots less than 50 m apart. Because these plots are near neighbors, they have a similar distance to the forest; for 75% of the plots, the difference in distance to the nearest forest was less than 50 m.

We propose associational effects mediated by intraspecific variation in neighbor quality for an ungulate; these correlative results should be confirmed by manipulative studies. Although most associational effect studies have assessed the impact of neighbors at close range (Champagne et al., 2016), our results suggest that the quality of neighboring plants could influence selection by deer at scales of tens to hundreds meters. Associational effect studies in forested environments often focus on maintaining a species negatively impacted by herbivory (Herfindal et al., 2015; Perea & Gil, 2014; Torroba‐Balmori, Zaldívar, Alday, Fernández‐Santos, & Martínez‐Ruiz, 2015). Managers cannot easily influence neighboring plant nutritional quality in most circumstances, but acknowledging such effects could help prioritize plantation or conservation efforts in overbrowsed landscapes. For example, planting of balsam fir among existing natural regeneration on Anticosti Island could take advantage of the neighboring plant nutritional quality. This could be performed using a forage nutritional quality map (Jean, Bradley, Tremblay, & Côté, 2015), where geostatistic techniques are used to interpolate value of forage quality on the landscape.

## AUTHORS' CONTRIBUTION

EC, SDC, and JPT conceived the project. EC collected and analyzed the data, led the interpretation and writing of the manuscript. SDC, BM, and JPT contributed to the interpretation. All authors contributed critically to the drafts and gave final approval for publication.

## DATA ACCESSIBILITY

Data will be deposited in the Dryad Digital Repository.

## CONFLICT OF INTEREST

None declared.
