# Supplementary material for: Spatial correlations between browsing on balsam fir by white‐tailed deer and the nutritional value of neighboring winter forage
Source: Ecol Evol. 2018 Feb 10;8(5):2812–23. doi: 10.1002/ece3.3878 (PMC5838068; doi:10.1002/ece3.3878)
Supplement: Supplementary file 1 [file ECE3-8-2812-s001.docx]

**Appendix S1. Parameters of calibration models from Near infrared reflectance spectroscopy**

**Table S1** Summary information of modified partial least squares regression models predicting fibre content on dry matter basis (%;NDF: hemicellulose, cellulose and lignin, ADF: cellulose and lignin, ADL: lignin), *in vitro* true digestibility on dry matter basis (IVTD_DM_) and nitrogen content, all expressed as percentages using Near infrared reflectance spectra (NIRS). The *r*^2^ value refers to the regression between laboratory and predicted values of the independent validation set. All regression models include values from different tree species: balsam fir (*Abies balsamea*), white spruce (*Picea glauca*), black spruce (*Picea mariana*) and paper birch (*Betula papyrifera*). The wavelength range used is 1100-2498.2 nm for all samples.

| Constituent | Value of lab analysis | Calibration set size | SECV^1^ | Mathematical treatment applied^2^ | Scatter correction | Validation set size | *r*^2^ |
| --- | --- | --- | --- | --- | --- | --- | --- |
|  | (Mean ± SD) |  |  |  |  |  |  |
| NDF | 37 ± 8 | 87 | 2.71 | 1,4,4,1 | None | 38 | 0.87 |
| ADF | 31 ± 6 | 88 | 2.53 | 2,4,4,1 | None | 38 | 0.80 |
| ADL | 17 ± 4 | 88 | 1.82 | 2,4,4,1 | None | 38 | 0.72 |
| IVTD_DM_ | 69 ± 8 | 65 | 2.75 | 1,4,4,1 | None | 29 | 0.88 |
| Nitrogen | 1.4 ± 0.7 | 110 | 0.10 | 1,4,4,1 | SNV^3^ | 46 | 0.97 |

^1^Standard error of cross-validation

^2^The first number is the derivative used, the second is the gap over which the derivative is calculated and the last two the degrees of primary and secondary smoothing. See DeGabriel et al. (2009) appendix for details.

^3^Standard normal variate (Barnes et al. 1989)

Reference**s**

Barnes R, Dhanoa M, Lister SJ (1989) Standard normal variate transformation and de-trending of near-infrared diffuse reflectance spectra. Appl Spectrosc 43:772-777

DeGabriel JL, Moore BD, Foley WJ, Johnson CN (2009) The effects of plant defensive chemistry on nutrient availability predict reproductive success in a mammal. Ecology 90:711-71

**Appendix S2. Additionnal cross-correlograms of the relation between browsing rate on balsam fir, and the abundance and nutritional quality of white spruce, balsam fir and paper birch, and correlations between nutritional quality variables**


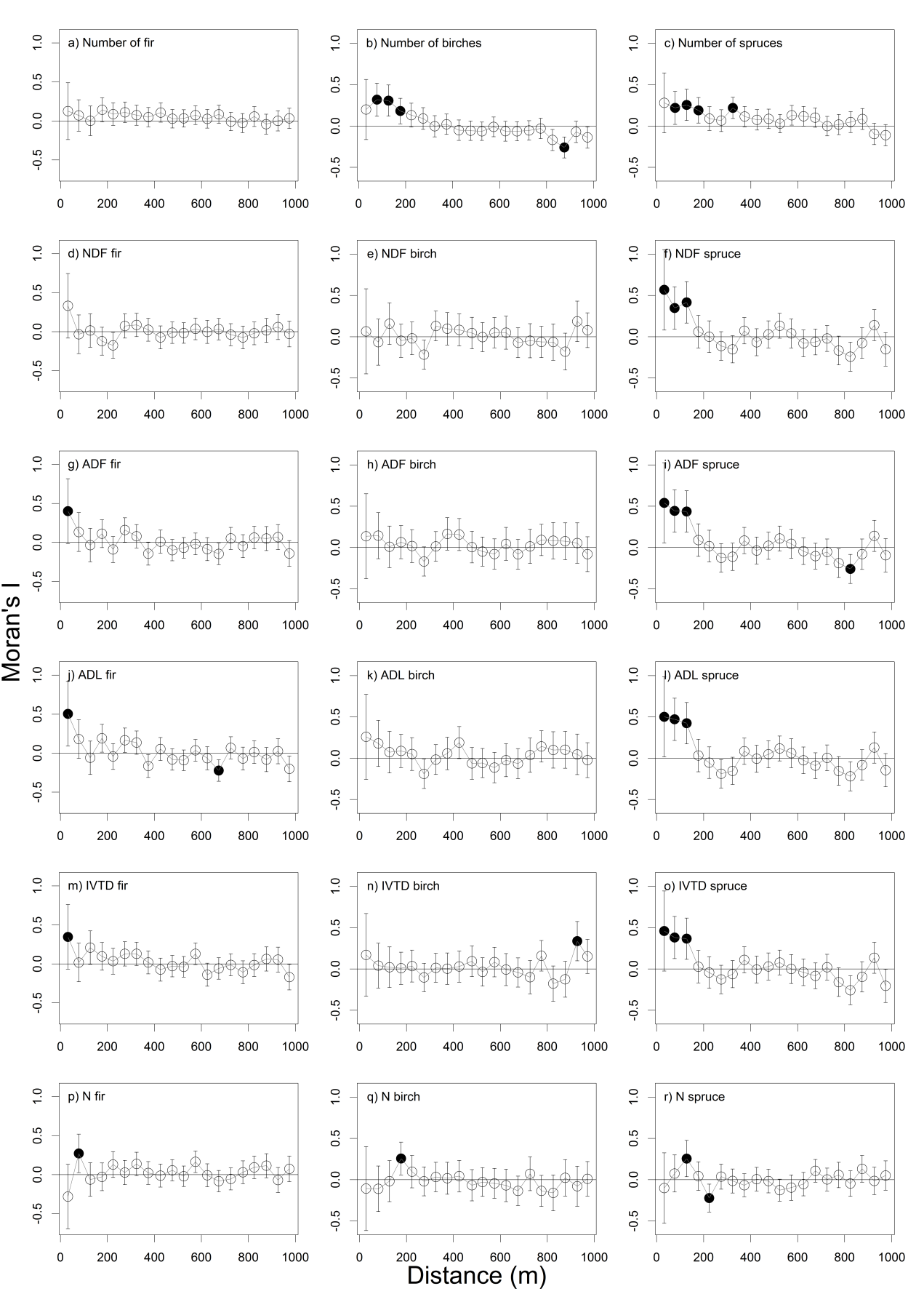
Figure S1. Moran’s I correlogram for the abundance and concentration of nutritional characteristics of balsam fir (first column), paper birch (second) and white spruce (third) in 40 m^2^ plots on Anticosti island (Québec, Canada). Nutritional characteristics include fibre content (NDF: hemicellulose, cellulose and lignin, ADF: cellulose and lignin, ADL: lignin), in vitro true digestibility on dry matter basis (IVTDDM) and nitrogen content (N). Moran’s I were calculated for pairs of plots in distance classes of 50 m and the point is located at the mean value for the class. The first bin included distance from 11 m to 50 m, since plots were separated by a minimum of 11 m to prevent overlap. Error bars are 95% confidence intervals. Black dots indicate statistically significant values with a progressive Bonferonni correction of the α-level, starting with α = 0.05


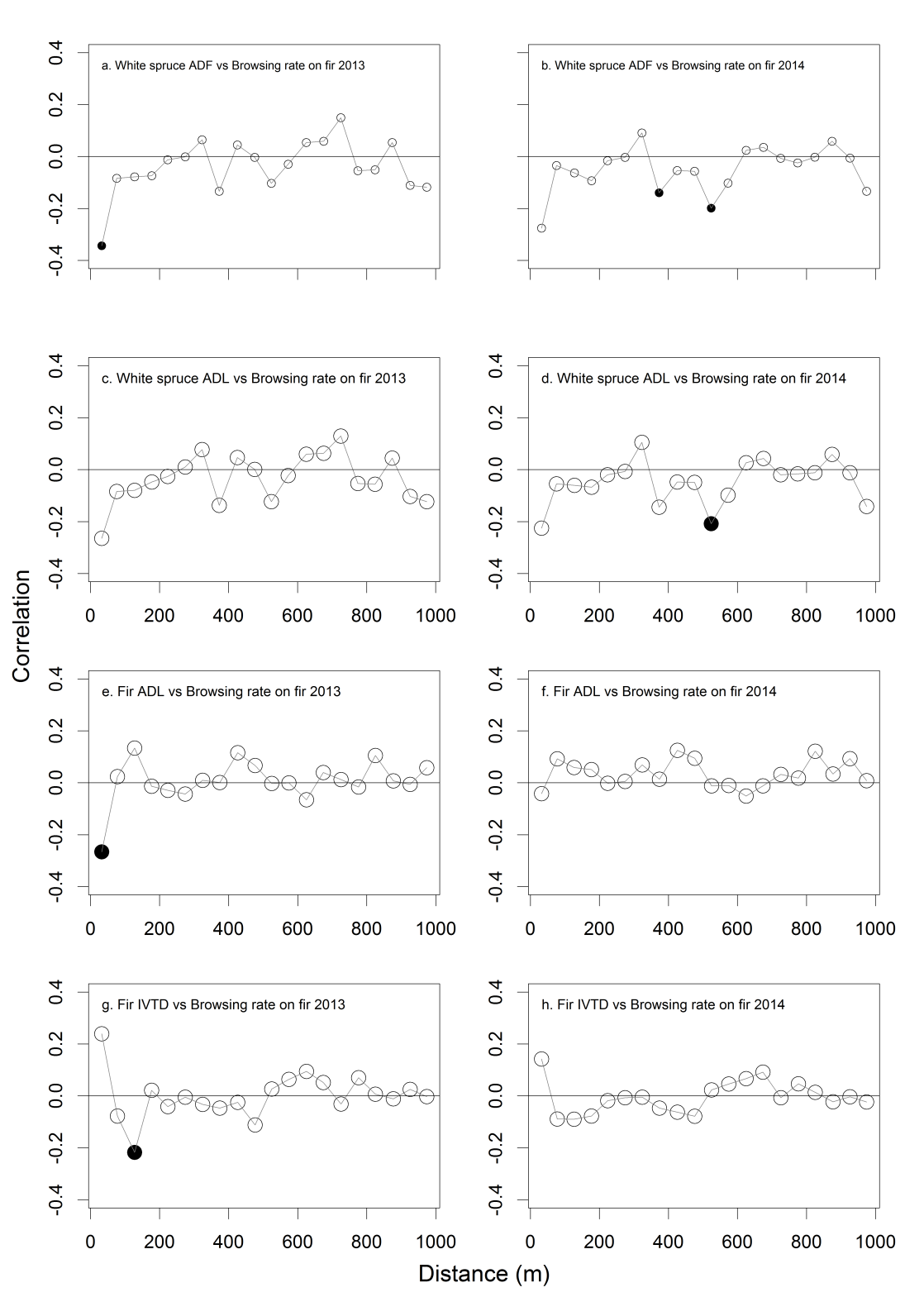
Figure S2 Cross-correlograms of the correlation between browsing rate on balsam fir (number of shoots browsed/number of shoots available) in 4 m2 plots in 2013 (left column) and 2014 (right column) and nutritional attributes of neighbouring white spruce (a-d) and fir (e-h). Nutritional characteristics include fibre content (ADF: cellulose and lignin, ADL: lignin) and in vitro true digestibility on dry matter basis (IVTDDM). Data were collected on Anticosti island (Québec, Canada). Correlations between each pair of variables were calculated for pairs of plots in distance classes of 50 m and the point is located at the mean value for the class. The first bin included distance from 11 m to 50 m, since plots were separated by a minimum of 11 m to prevent overlap. Black dots indicate statistically significant values with a progressive Bonferonni correction of the α-level, starting with α = 0.05.


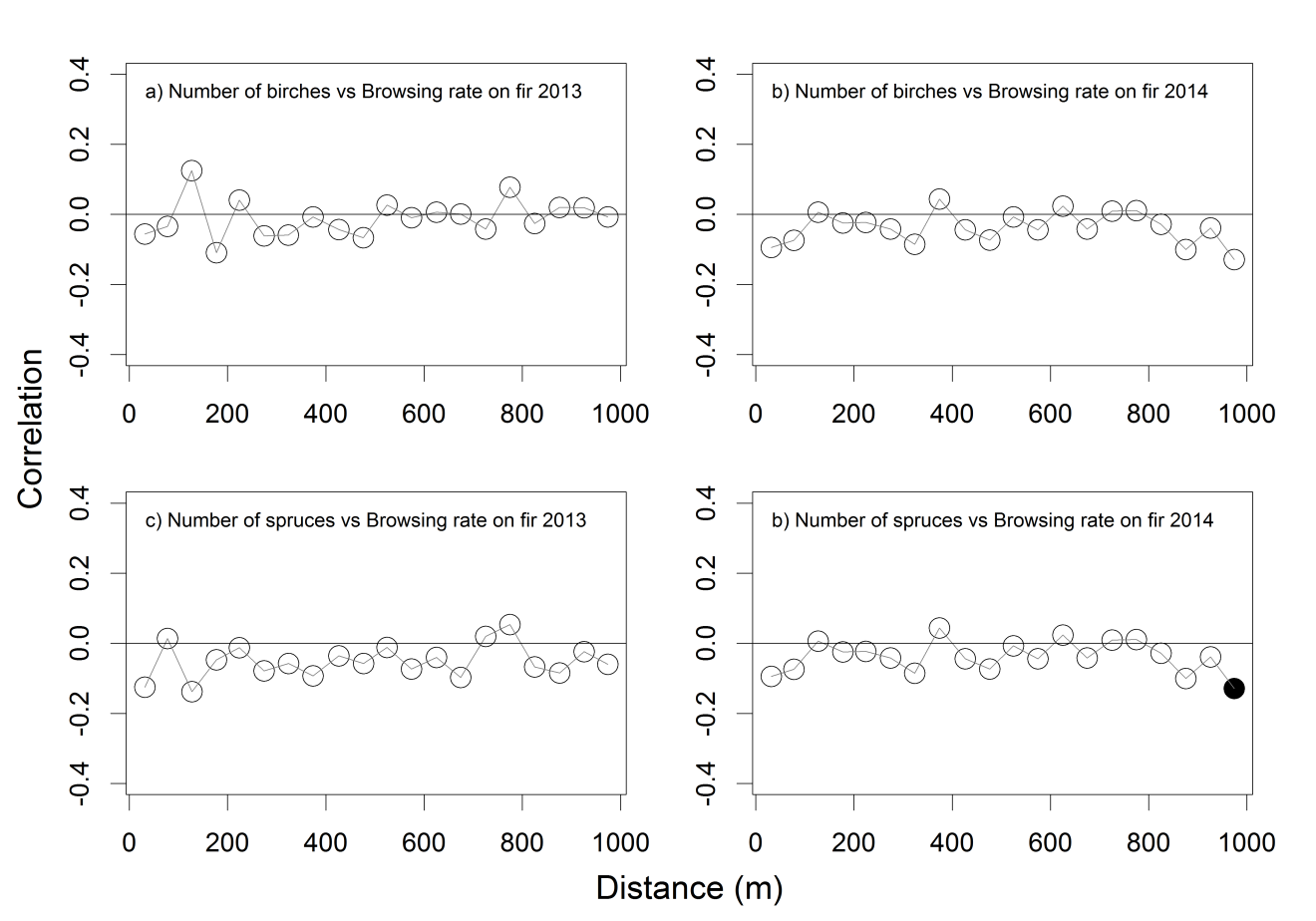
Figure S3 Cross-correlograms of the correlation between browsing rate on balsam fir (number of shoots browsed/number of shoots available) in 4 m^2^ plots in 2013 (left column) and 2014 (right column), and the number of paper birches (a-b) and white spruces (c-d) in 40 m^2^ plots. Data were collected on Anticosti island (Québec, Canada). Correlations between each pair of variables were calculated for pairs of plots in distance classes of 50 m and the point is located at the mean value for the class. The first bin included distance from 11 m to 50 m, since plots were separated by a minimum of 11 m to prevent overlap. Black dots indicate statistically significant values with a progressive Bonferonni correction of the α-level, starting with α = 0.05.

Table S2 Correlations between nutritional attributes of balsam fir, white spruce and birch inside the 4 m^2^ plots. Nutritional characteristics include fibre content (NDF: hemicellulose, cellulose and lignin, ADF: cellulose and lignin, ADL: lignin), in vitro true digestibility on dry matter basis (IVTDDM) and nitrogen content (N). Data were collected on Anticosti island (Québec, Canada).We calculated correlation values with the cor.test function of R 3.2.1 ([R Core Team 2015](#_ENREF_71)) and we report t values, p-values and the r value for the correlation. Significant p-values at α = 0.10 are in bold.

|  | **Fir and spruce** | **Fir and birch** | **Spruce and birch** |
| --- | --- | --- | --- |
| **NDF** | t_70_ = 0.36 p = 0.72 r = 0.04 | t_62_ = 0.12 p = 0.91 r = 0.01 | t_47_ = 0.51 p = 0.61 r = 0.07 |
| **ADF** | t_70_ = 2.59 **p = 0.01** r = 0.30 | t_62_ = -0.51 p = 0.61 r = -0.07 | t_47_ = 0.14 p = 0.89 r = 0.02 |
| **ADL** | t_70_ = 1.74 **p = 0.09** r = 0.20 | t_62_ = -0.52 p = 0.60 r = -0.07 | t_47_ = 0.22 p = 0.82 r = 0.03 |
| **IVTD** | t_70_ = 0.70 p = 0.49 r = 0.08 | t_62_ = -0.45 p = 0.65 r = -0.06 | t_47_ = 0.90 p = 0.37 r = 0.13 |
| **N** | t_70_ = 2.27 **p = 0.03** r = 0.26 | t_62_ = -0.06 p = 0.95 r = -0.01 | t_47_ = -3.02 **p = 0.004** r = -0.40 |
